# Treatment of Sternocutaneous Fistula Due to Cardiac Surgery Using Extracellular Matrix Patch

**DOI:** 10.21470/1678-9741-2024-0137

**Published:** 2025-04-28

**Authors:** Zoran Tabaković, Milana Marinković, Petar Milačić1, Slobodan Mićović, Igor Živković

**Affiliations:** 1 Cardiac Surgery Clinic, Institute for Cardiovascular Diseases “Dedinje”, Belgrade, Serbia; 2 Clinic for Burns, Plastic, and Reconstructive Surgery, University Clinical Center of Serbia, Belgrade, Serbia; 3 School of Medicine, University of Belgrade, Belgrade, Serbia

**Keywords:** Incidence, Sternum/surgery, Surgical Wound Infection, Cutaneous Fistula, Cardiac Surgical Procedures

## Abstract

The incidence of sternal wound complications, such as dehiscences, infections, and sternocutaneous fistulas, can reach 10%. Sternocutaneous fistulas are extremely rare, and the only definite therapy is surgical repair. Our experience taught us that combining a traditional approach with an extracellular matrix patch might be a step forward in therapy. We described three examples of surgically reconstructing sternocutaneous fistulas with an extracellular matrix patch (ProxiCor®).

## INTRODUCTION

**Table:** 

Abbreviations, Acronyms & Symbols	
CABG	= Coronary artery bypass grafting
SCFs	= Sternocutaneous fistulas
SSIs	= Surgical site infections
VAC	= Vacuum-assisted closure

One of the most frequent complications of cardiac surgery is surgical site infections (SSIs), which are most common in the sternal wound region^[1]^. The reported incidence is up to 9.7%, with 1-3% of patients developing severe sternal wound infection^[1]^. Most SSIs occur during the first few weeks after cardiac surgery. On the other hand, it may be diagnosed months or even years later, as is the case with chronic sternocutaneous fistulas (SCFs), which are infrequent complications^[1]^. The treatment protocol includes administering antibiotics combined with negative pressure (vacuum-assisted closure [VAC] system), and different surgical techniques for reconstruction. Titanium plates, biodegradable implants, and allogenic sternal allografts are all viable options for surgical reconstruction^[2]^.

Here we presented three patients with SCFs who underwent surgical repair using an extracellular matrix patch (ProxiCor®).

## CASE PRESENTATION

### First Patient

In January 2019, a 69-year-old female patient with a history of coronary artery disease and type 1 diabetes was admitted to our hospital and underwent coronary artery bypass grafting (CABG) revascularization. The surgical procedure and the early postoperative period were uneventful. Three weeks following the surgery, the patient was readmitted due to a presternal infection, which was treated conservatively using systemic antibiotics. After one month, a negative microbiological culture was discovered, and debridement with pectoral plastic was performed. The postoperative phase was uneventful, and the patient was released home.

**Fig. 1:**
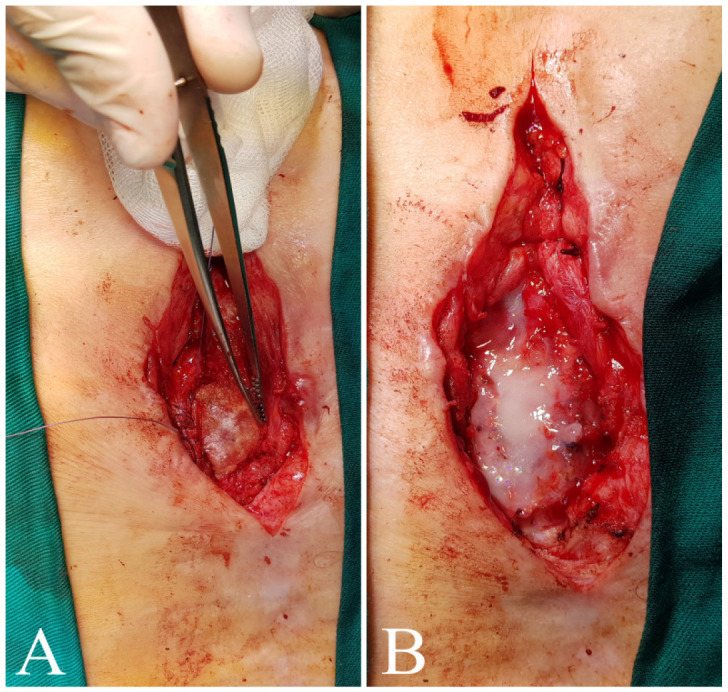
Surgical reconstruction of sternocutaneous fistula in the first patient. A) Debridement and excision of the fistula, defect filled with vancomycin paste and extracellular matrix patch. B) Defect covered with fibrine glue.

Several weeks later, the patient was admitted again, this time with a SCF in the lower part of the sternal wound filled with pus. *Pseudomonas aeruginosa* and *Staphylococcus aureus* were isolated from the wound swab. Debridement and excision of the fistula in the xiphoid part of the sternum were done, while the newly created defect was filled with vancomycin paste and extracellular matrix patch (ProxiCor®) (Figure 1A). The place of the defect was covered with fibrine glue, and the wound was closed by anatomic layers with single PDS® stitches (Figure 1B). Postoperative period and one-month local findings were both regular. Five-year follow-up has shown no recidivism after treatment.

### Second Patient

In September 2018, a 47-year-old male patient was admitted to our hospital and underwent CABG revascularization. The patient had a previous history of myocardial infarction and coronary artery disease and a low ejection fraction (35%). The surgical technique, early postoperative phase, and subsequent evaluations were unremarkable.

**Fig. 2:**
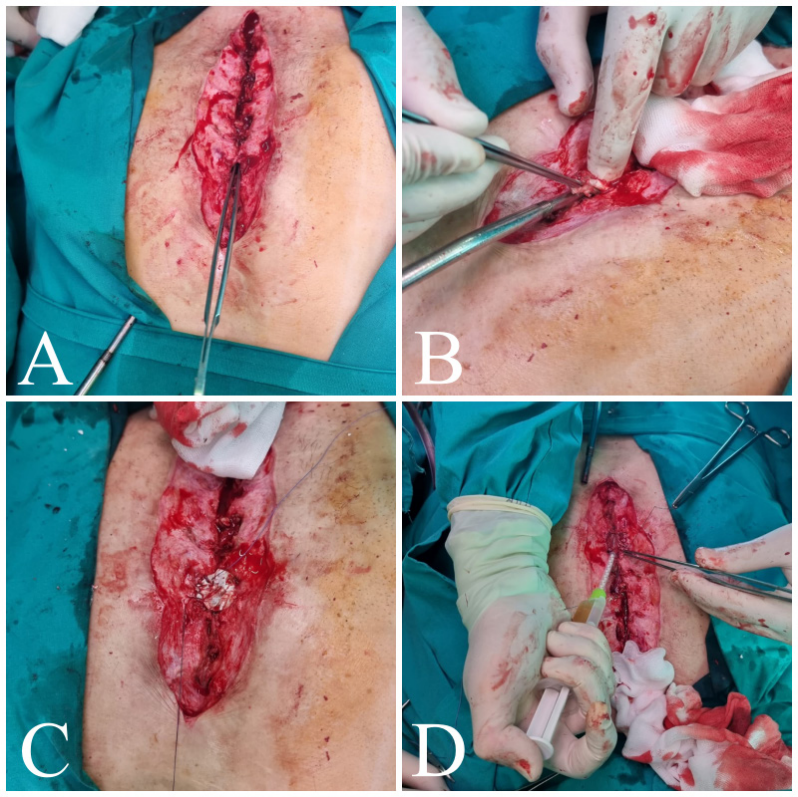
Surgical reconstruction of sternocutaneous fistula in the second patient. A) Detecting fistulas in the median portion of the sternum. B) Debridement of wound and resection of fistula, defect filled with extracellular matrix patch and vancomycin paste. C) Extracellular matrix patch sutured to the surrounding tissue. D) Defects covered with BioGlue®.

In June 2023, two SCFs developed in the sternal region with *Staphylococcus aureus* in microbiological culture. Additionally, the patient acquired grade 5 renal failure and required daily hemodialysis therapy. He was hospitalized, and several days later, we performed surgical procedures for SCFs. Following the typical preoperative protocol and surgical preparation, the incision was opened through all anatomical layers of the presternal region, revealing metal osteosynthesis stitches and two fistulas in the median portion of the sternum (Figure 2A). Metal stitches were removed, the presternal wound was debrided, and the fistula was resected. Extracellular matrix patches (ProxiCor®) and vancomycin paste were used to fill newly formed defects (Figure 2B). The extracellular matrix patch was sutured to the surrounding tissue (Figure 2C). The filled defects were covered using BioGlue®, and the wound was sutured with single PDS® stitches across anatomical layers (Figure 2D). The postoperative course was regular. Two months later, an examination showed that the wound was healing adequately. Six-month after-treatment follow-up has shown no recidivism, but unfortunately, eight months after treatment, the patient died because of multiple myeloma.

### Third Patient

In March 2023, a 66-year-old male patient was admitted to our hospital undergoing an elective CABG procedure. The medical history revealed developed coronary artery disease and diabetes mellitus type 2. We used both right and left mammary arteries and one venous saphenous graft for coronary revascularization. The CABG treatment followed a standard postoperative course, and the patient was discharged to home recovery.

**Fig. 3:**
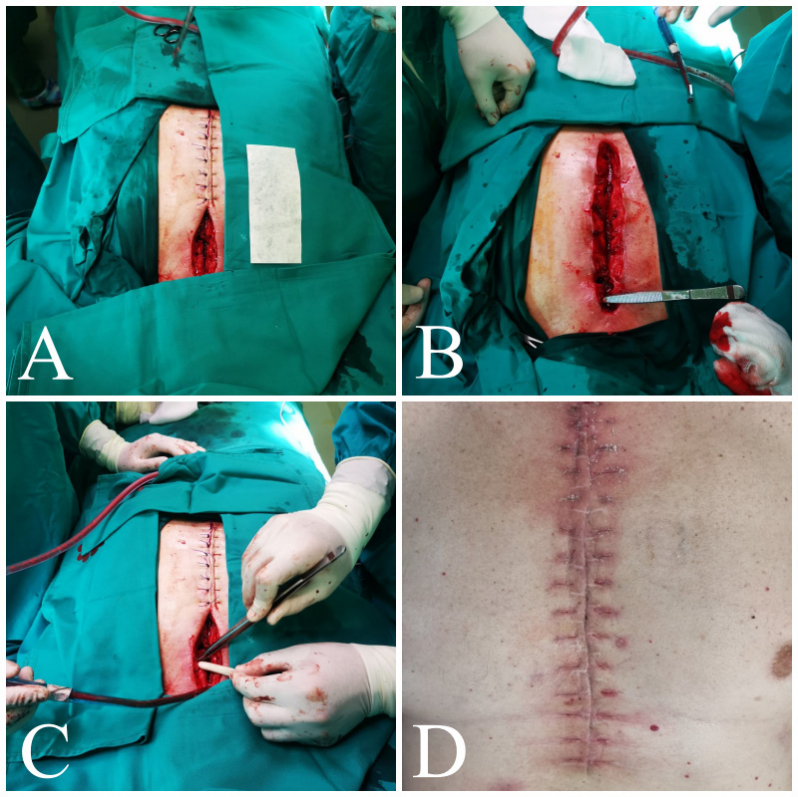
Surgical reconstruction of sternocutaneous fistula in the third patient. A) The lower half of the incision was sutured with single PDS® stitches. B) Identification of a fistula in the upper section of the incision. C) The defect is filled with vancomycin paste and an extracellular matrix patch. D) Examination control after one month.

The patient was readmitted in August 2023 due to wound dehiscence and SCF in the upper sternum. Following the typical preoperative protocol, the surgical reconstruction was performed. Following presternal debridement, the lower half of the incision was sutured with single PDS® stitches by anatomical layers (Figure 3A). We identified a fistula in the upper section of the incision that extended to the left pectoralis muscle (Figure 3B). We excised the fistulous tissue and filled the defect with vancomycin paste and an extracellular matrix patch (ProxiCor®) (Figure 3C). The rest of the incision was sutured with single PDS® stitches through anatomical layers. The postoperative course was regular, and examination controls after two weeks and one month showed a regular healing process (Figure 3D). Eight-month after-treatment follow-up has shown no signs of recidivism.

## DISCUSSION

Extracellular matrix patches are relatively novel biomaterials that can be extremely useful in tissue repair and regeneration, therefore they might be widely used in surgery, particularly in cardiac surgery to repair injured cardiac and vascular tissues^[3]^. Given the repair feature, our primary goal in treating chronic sternal wounds was to promote the natural healing process of conductive tissues, thus accelerating patients' recovery following reconstructions and reducing their chance of recidivism.

Despite all precautions, sternal wound complications and infections are not so rare, and various treatment options are available, each of which requires time and patience^[1^,^4]^. SCFs are infrequent sternal wound complications with a high risk of recurrence, treatment of this condition is very complex, and the surgeon must have experience in reconstructive surgery^[5]^. SCF can be life-threatening, and some of these individuals require significant surgical therapy, including muscle or omentum flap reconstruction. The morbidity and mortality increase hospital stay. Negative pressure wound therapy (VAC), either alone or combined with antibiotic and surgical therapy, can be an effective treatment option in some cases.

Although not commonly used in reconstructive surgery for sternal wound problems, the use of an extracellular matrix patch might be a step forward in treating these patients. To our knowledge, no medical literature mentions using extracellular matrix patches in treating SCFs. Boulemden et al.^[6]^ published one case report in which they employed an extracellular matrix patch to treat sternal wound dehiscence in newborns following heart surgery. Just as in our situation, it produced positive benefits in the healing process, with patients recovering significantly faster and in-hospital stays substantially shorter.

## CONCLUSION

Although there is a lack of literature on this topic, our case report series demonstrated that using an extracellular matrix patch could be very useful in treating patients with chronic wound complications such as SCFs. However, using an extracellular matrix patch alone in treatment cannot be a simple and definitive solution. Our experience has shown that combining an extracellular matrix patch with a standard approach may be a step forward in the treatment of SCFs, but further research is needed because of a lack of topics on the subject.
